# Diagnostic Sensitivity of Blood Culture, Intraoperative Specimen, and Computed Tomography-Guided Biopsy in Patients with Spondylodiscitis and Isolated Spinal Epidural Empyema Requiring Surgical Treatment

**DOI:** 10.3390/jcm12113693

**Published:** 2023-05-26

**Authors:** Mido Max Hijazi, Timo Siepmann, Alexander Carl Disch, Uwe Platz, Tareq A. Juratli, Ilker Y. Eyüpoglu, Dino Podlesek

**Affiliations:** 1 Department of Neurosurgery, Division of Spine Surgery, Technische Universität Dresden, Faculty of Medicine, and University Hospital Carl Gustav Carus, Fetscherstrasse 74, 01307 Dresden, Germany; tareq.juratli@ukdd.de (T.A.J.); ilker.eyuepoglu@ukdd.de (I.Y.E.); dino.podlesek@ukdd.de (D.P.); 2 Department of Neurology, Technische Universität Dresden, Faculty of Medicine, and University Hospital Carl Gustav Carus, Fetscherstrasse 74, 01307 Dresden, Germany; timo.siepmann@ukdd.de; 3 Department of Orthopedics and Traumatology, Technische Universität Dresden, Faculty of Medicine, and University Hospital Carl Gustav Carus, Fetscherstrasse 74, 01307 Dresden, Germany; alexander.disch@ukdd.de (A.C.D.); uwe.platz@ukdd.de (U.P.)

**Keywords:** spondylodiscitis, isolated spinal epidural empyema, diagnostic sensitivity, blood culture, intraoperative specimen, computed tomography-guided biopsy

## Abstract

Background: the successful treatment of spondylodiscitis (SD) and isolated spinal epidural empyema (ISEE) depends on early detection of causative pathogens, which is commonly performed either via blood cultures, intraoperative specimens, and/or image-guided biopsies. We evaluated the diagnostic sensitivity of these three procedures and assessed how it is influenced by antibiotics. Methods: we retrospectively analyzed data from patients with SD and ISEE treated surgically at a neurosurgery university center in Germany between 2002 and 2021. Results: we included 208 patients (68 [23–90] years, 34.6% females, 68% SD). Pathogens were identified in 192 cases (92.3%), including 187 (97.4%) pyogenic and five (2.6%) non-pyogenic infections, with Gram-positive bacteria accounting for 86.6% (162 cases) and Gram-negative for 13.4% (25 cases) of the pyogenic infections. The diagnostic sensitivity was highest for intraoperative specimens at 77.9% (162/208, *p* = 0.012) and lowest for blood cultures at 57.2% (119/208) and computed tomography (CT)-guided biopsies at 55.7% (39/70). Blood cultures displayed the highest sensitivity in SD patients (SD: 91/142, 64.1% vs. ISEE: 28/66, 42.4%, *p* = 0.004), while intraoperative specimens were the most sensitive procedure in ISEE (SD: 102/142, 71.8% vs. ISEE: 59/66, 89.4%, *p* = 0.007). The diagnostic sensitivity was lower in SD patients with ongoing empiric antibiotic therapy (EAT) than in patients treated postoperatively with targeted antibiotic therapy (TAT) (EAT: 77/89, 86.5% vs. TAT: 53/53, 100%, *p* = 0.004), whereas no effect was observed in patients with ISEE (EAT: 47/51, 92.2% vs. TAT: 15/15, 100%, *p* = 0.567). Conclusions: in our cohort, intraoperative specimens displayed the highest diagnostic sensitivity especially for ISEE, whereas blood cultures appear to be the most sensitive for SD. The sensitivity of these tests seems modifiable by preoperative EAT in patients with SD, but not in those with ISEE, underscoring the distinct differences between both pathologies.

## 1. Introduction

Spondylodiscitis (SD) and isolated spinal epidural empyema (ISEE) are common types of primary spinal infections that are challenging to treat [1,2,3]. The reported incidence in the literature is five to six cases per 100,000 patient-years, but recent data suggests a higher incidence of 30/250,000 [4]. ISEE is an isolated infection of the epidural space with the accumulation of purulent substance without a concurrent SD, while SD refers to a primary infection of the intervertebral disc with secondary osteomyelitis of the adjacent endplates that occasionally occurs with epidural empyema [5,6].

Standardized treatment guidelines for SD and ISEE are currently lacking [7,8]. Surgical treatment followed by conservative management with antibiotic therapy takes several weeks and months for patients to recover. However, inadequate antibiotic therapy during this period can increase patient morbidity [9]. Reliable diagnostics including pathogen detection with antibiogram resistogram are indispensable for infection treatment. Three procedures are available for isolating causative pathogens, including blood cultures, intraoperative specimens, and computed tomography (CT)-guided biopsies.

Some studies suggest that blood cultures can yield early positive results due to hematogenous spreading of the infection, in particular in patients with SD [10]. To minimize a contamination, blood cultures need to be withdrawn from two or three different sites [11]. At least three pairs of blood cultures (aerobic and anaerobic culture medium) should be collected before the initiation of antibiotic therapy, irrespective of febrile temperatures [12]. This allows the isolation of a causative pathogen in up to 37.5% of cases [13].

Intraoperative sampling on site of the infectious focus is considered the most sensitive and specific method for pathogen detection [14]. CT-guided biopsy is an additional option to isolate pathogens, especially in cases when abscess formations such as psoas abscesses are visible on diagnostic imaging. However, the sensitivity of each procedure is limited when performed alone, whereas a combination of these procedures can increase the likelihood of successful pathogen detection [15,16].

In case of severe infections, empirical antibiotic therapy (EAT) is usually initiated preoperatively and then switched to targeted antibiotic therapy (TAT) after the causative pathogen has been identified, based on resistogram. Other cases are initially managed with TAT according to the resistogram [4].

A few studies have reported on the diagnostic sensitivity of these three procedures for SD and ISEE [12,17]. However, the impact of ongoing EAT on pathogen detection in SD and ISEE has not been thoroughly addressed. Therefore, this retrospective study aims to assess the diagnostic sensitivity of the three procedures and evaluate the influence of prior EAT on outcomes.

## 2. Materials and Methods

### 2.1. Study Design and Patient Data

A retrospective observational study was performed on a cohort of 208 consecutive patients with SD and ISEE who underwent surgical treatment at our neurosurgery department from 2002 to 2021. All surgically treated patients with SD or ISEE aged over 18 years and without intradural infection were included. The study was approved by the local ethics committee of Dresden university hospital (Reference number BO-EK-17012022). Patient data were identified and extracted via review of electronic medical records using the ORBIS system (ORBIS, Dedalus, Bonn, Germany) and neuroimaging files through the IMPAX system (IMPAX, Impax Asset Management Group plc, London, UK). Detailed demographic, clinical, radiological, laboratory and microbiological analyses were evaluated between groups.

### 2.2. Clinical Management

The diagnosis of SD or ISEE was obtained according to the Clinical Practice Guidelines for the diagnosis and treatment of native vertebral osteomyelitis in Adults of the Infectious Diseases Society of America (IDSA) [18]. Primary treatment was conservative with intravenous antibiotics in a conservative external or internal clinic, whereas the patient was referred to our clinic if surgical treatment was required, e.g., in cases with neurological deficits, spinal instability, or epidural abscess. Our cohort of 187 patients included surgical ISEE or SD patients with primary immediate indication for open surgery. Depending on disease severity, surgical therapy involved abscess evacuation, dorsal decompression with/without dorsal interbody fusion, ventral debridement with anterior cervical discectomy and fusion (ACDF), or vertebral body replacement. Surgical decision making was based on clinical experience and several defined radiographic signs. Patients undergoing spinal instrumentation had preoperative CT scans to assess bony integrity.

Patients with ISEE were treated with abscess evacuation with/without drainage or ACDF for abscesses ventral to the cervical spinal cord. Patients with SD underwent either abscess evacuation or/and instrumentation for instability, deformity, and pain-related immobility. In case of psoas abscess, drainage was performed CT-guided.

SD patients with a spinal epidural abscess without deformity underwent decompression with removal of the abscess and vertebral disc with/without dorsal interbody fusion. If the vertebral body height reduction was less than 50%, dorsal decompression with interbody fusion was performed first, and optionally with secondary ventral debridement with vertebral body replacement. In cases where vertebral body reduction was more than 50%, dorsal decompression with instrumentation followed by vertebral body replacement was performed.

### 2.3. Antibiotic Therapy

Each patient received either TAT or EAT, depending on the clinical condition at the time of admission and as recommended by the local infectious disease department. Our cohort included many patients with known infection who were already treated with antibiotics externally and presented to our clinic for surgical treatment. If antibiotics were continued or discontinued for less than 48 h, we defined this condition as being under ongoing antibiotic therapy and called this group EAT, whereas patients without antibiotic therapy or with an antibiotic-free period of more than 2 days formed the TAT group. EAT was switched to TAT after the identification of the pathogen. Intravenous antibiotic therapy was switched to oral antibiotics after approximately 4 weeks, and the total duration of antibiotic therapy was approximately 8 weeks.

### 2.4. Methods for Pathogen Detection

Blood cultures were preferably collected from all patients before starting antibiotic therapy, using three pairs of blood cultures at two or three different peripheral sites (aerobic and anaerobic media) for microbiological assessment. Some patients presented to our clinic in a septic condition with ongoing antibiotic therapy, thus blood culture collection in an antibiotic-free period was not possible, nor was antibiotic suspension justifiable in this case.

Intraoperatively obtained tissue was placed in Schaedler boullions (bioMérieux, Nürtingen, Germany) directly in the operating room and these were then sent to our institute of medical microbiology and virology for analysis. There, the boullions were first incubated at 37 degrees and examined for turbidity after 48 h. Once turbidity was detected, the culture suspension was plated out on Columbia Blood Agar (bioMérieux, Nürtingen, Germany) and HCB Agar (bioMérieux, Nürtingen, Germany). Aerobic culture growth was checked for the first time after 24 h and anaerobic culture growth after another 48 h.

CT-guided biopsy was performed exclusively in case of psoas abscess by the neuroradiologists or radiologists, whereas paravertebral dorsal abscesses and epidural empyema were managed during open surgery. All patients received a pre-interventional contrast-enhanced MRI of the spine and an additional CT for planning. A psoas abscess was always observed on contrast-enhanced MRI of the spine and classified by the neuroradiologist or radiologist as a formation with fluid-equivalent signal intensity and biopsy-worthy formation. An abscess was defined as iso- or hypointense on T1-weighted images, with fluid-equivalent signal intensity on T2-weighted images, and with edge enhancement on contrast-enhanced T1-weighted fat-saturated images [19]. Our cohort did not include CT-guided biopsy of disc, vertebral body, dorsal paravertebral abscess, or epidural abscess. The approach was chosen based on anatomic considerations and the predominant site of the infective lesions. The liquefied contents of the abscess were aspirated and sent for microbiological examination; in addition, a sample was fixed in formalin for pathohistological analysis. In our study, 128 patients (61.5%) were diagnosed with psoas abscess, of which 70 patients (54.7%) were tappable, 50 patients had SD, and 20 had ISEE. Following CT-guided sampling, all patients received a suction-irrigation drainage system from which samples were collected two times daily, and the abscess cavity was also irrigated with gentamycin or vancomycin two times daily, depending on the resistogram. After obtaining three pathogen-free results from the suction-irrigation drainage samples, the drainage was removed.

### 2.5. Microbiological Assessment

Bacteria with high to moderate pathogenic potential that are unlikely to present as contaminants, such as methicillin-susceptible staphylococcus aureus (MSSA), were deemed significant if they were detected in at least one culture. However, potentially low pathogenic bacteria, e.g., cutibacterium, were only considered clinically significant if they were identified in at least two independent cultures. Cases with negative microbiologic results, but with clear clinical and radiographic evidence of SD or ISEE, were classified as positive with non-identified pathogens.

### 2.6. Case Presentation

**Figure 1:**
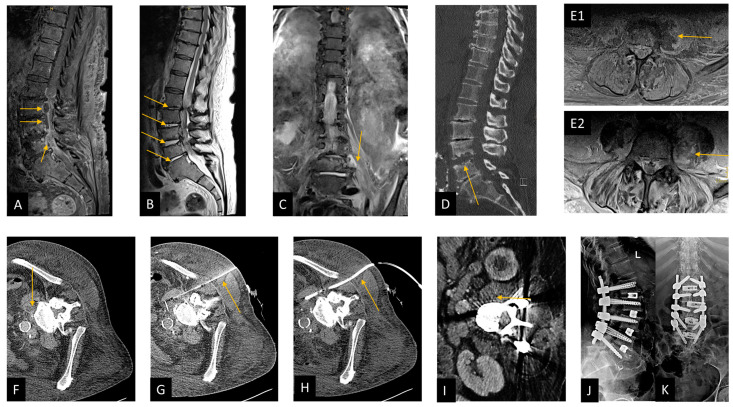
Case presentation demonstrating CT-guided biopsy and surgical management in spondylodiscitis. This figure shows a patient from our cohort who suffered from spondylodiscitis with concomitant spinal epidural empyema and psoas abscess left; the 74-year-old patient was pretreated externally with calculated antibiotics (ceftriaxone, flucloxacillin, and metronidazole) and had paraparesis of the legs and sepsis; risk factors were a BMI greater than 35 kg/m and diabetes mellitus with chronic malum perforans pedis. Blood cultures were initially obtained from two different peripheral regions, followed immediately with microsurgical decompression with abscess evacuation and application of a suction-irrigation drainage system. CT-guided drainage of the psoas abscess was performed on the first postoperative day, whereas *Staphelococcus aureus* was detected only in blood culture and subsequently treated with flucloxacillin and rifampicin. Due to increasing bone destruction, transforaminal lumbar interbody fusion (TLIF) was performed at L1/L2, L2/L3, L3/4, and L4/L5 level. The patient was moved from the intensive care unit to the normal ward and mobilized at ward level. (**A**) Preoperative sagittal T1-weighted fat-saturated contrast-enhanced MRI image of the lumbar spine shows the epidural abscess in the spinal canal at L2–L4 level, marked with arrows. (**B**) Preoperative sagittal T2-weighted MRI image, arrows show spondylodiscitis at L1/L2, L2/L3, L3/L4, and L4/L5 level. (**C**) preoperative coronal T2-weighted short-tau inversion recovery (T2w-STIR) MRI image, arrow shows a psoas abscess on the left. (**D**) Preoperative sagittal reformated CT image, arrow shows bone destruction mainly at the level of L4/L5. (**E1**) Preoperative axial T1-weighted fat-saturated contrast-enhanced MRI image, arrow shows psoas abscess. (**E2**) Preoperative axial T2-weighted fat-saturated MRI image with arrow pointing to psoas abscess on the left. (**F**–**H**): Illustration of CT-guided puncture of a left psoas abscess in three steps in an axial CT image. (**F**) Planning CT, (**G**) needle puncture, and (**H**) insertion of a suction-irrigation drain. Partially imaged central venous catheter in the iliac vein (**G**,**H**). (**I**) Postoperative axial CT image showing the regreening of the psoas abscess after draining the abscess. (**J**,**K**) A postoperative lateral (**J**) and anteroposterior (**K**) radiograph after performing TLIF spondylodesis from L1 to L5.

This following figure presented a case from our series showing our clinical management of spondylodiscitis (Figure 1).

### 2.7. Statistical Analysis

Data were statistically analyzed with the SPSS software package (SPSS Statistics 28, IBM, Armonk, New York, NY, USA). Descriptive statistics were used, and categorical variables were adjusted by Fisher exact tests or chi-square tests where appropriate. Numerical variables were analyzed with Mann-Whitney U tests. A binomial test was also used. All statistical tests were two-sided, and a value *p* < 0.05 was considered statistically significant. The sensitivity of a method was calculated as follows: S = true positive/(true positive + false negative) [20].

## 3. Results

### 3.1. Demographics and Baseline Characteristics

We enrolled 208 patients (males: 136, 65.4% vs. females: 72, 34.6%, *p* < 0.001) aged 68 [23–90] y, median [interquartile range] with SD (142, 68.3%) and ISEE (66, 31.7%). Intraoperative specimens and blood cultures were obtained in all cases, while computed tomography (CT)-guided biopsies were performed in 70 tappable cases (54.7%) of 128 patients with psoas abscess (61.5%). A causative pathogen was isolated in 192 cases (92.3%), of which 187 cases (97.4%) presented a pyogenic pathogen and five cases (2.4%) a non-pyogenic pathogen. Gram-positive pathogens were detected in 162 of 187 cases (86.6%), whereas a Gram-negative pathogen was identified in only 25 cases (13.4%). Empiric antibiotic therapy (EAT) was initiated preoperatively in 140 patients (67.3%) and switched following pathogen detection, whereas targeted antibiotic therapy (TAT) was started postoperatively according to resistogram in 68 patients (32.7%). Twelve patients (6%) died due to the disease and its complications (Table 1).

Ages, sex, diabetes mellitus, immunosuppression, and obesity are the most known risk factors in SD and ISEE. In our cohort, diabetes mellitus was observed in 70 patients (37.4%), while 59 patients (31.6%) had a BMI (body mass index) over 30 kg/m^2^ and 27 patients (14.4%) were immunosuppressed.

Primary sources of infection were identified in 137 patients (73.3%); however, infections resulting directly from surgical spine procedures were not included in this study. We identified 37 skin infections (19.8), 17 infections after epidural application (9.1%), 16 respiratory tract infections (8.6%), nine gastrointestinal tract infections (4. 8%), 13 urinary tract infections (7.0%), eight port-associated infections (4.3%), six retropharyngeal and prevertebral infections (3.2%), 22 foreign body-associated infections (11.8%), three endocarditis of prosthetic valves (1, 6%), five odontogenic infections (2.7%), one infection attributable to immunodeficiency (0.5%), while in 50 patients (26.7%) the cause of infection remained unclear.

### 3.2. Diagnostic Sensitivity of Procedures

**Figure 2:**
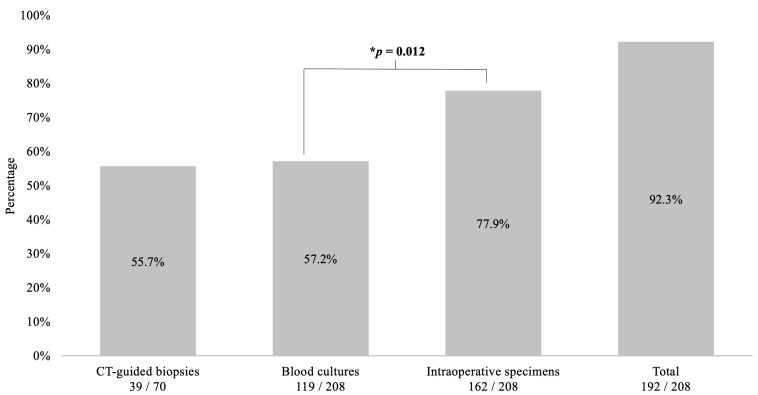
Sensitivity of blood culture, intraoperative specimen, and CT-guided biopsy. This figure shows the diagnostic sensitivity of the three procedures in combination and alone. The best results are obtained when all diagnostic procedures are used together, and the best individual result is achieved with intraoperative specimens, which are significantly more effective than blood cultures (77.9% vs. 57.2%, *p* = 0.012). * Binomial test. CT: computed tomography.

The highest sensitivity of pathogen detection was achieved using intraoperative specimens (162/208, 77.9%), followed by blood cultures (119/208, 57.2%) and being lowest at CT-guided biopsies (39/70, 55.7%, *p* = 0.012). The diagnostic sensitivity was 92.3% (192/208) for all procedures combined (Figure 2).

### 3.3. Diagnostic Sensitivity in SD and ISEE

**Figure 3:**
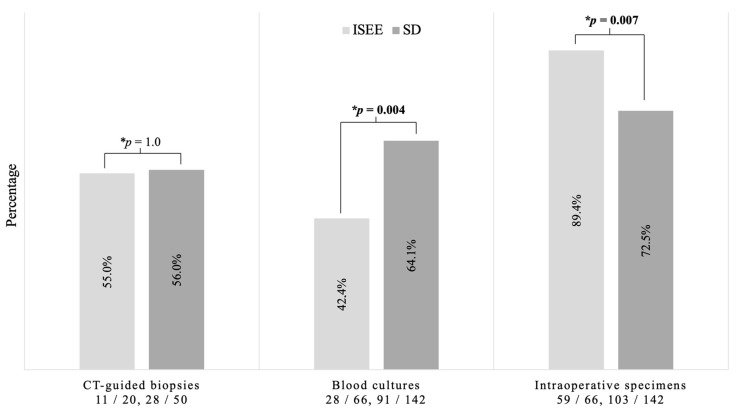
Diagnostic sensitivity in spondylodiscitis and isolated spinal epidural empyema. This figure shows the significant difference between both groups, especially in blood cultures (*p* = 0.004) and intraoperative specimens (*p* = 0.007). No difference was found between both groups concerning CT-guided biopsies. * Fisher exact test. ISEE: isolated spinal epidural empyema SD: spondylodiscitis, CT: computed tomography.

Blood cultures demonstrated significantly higher diagnostic sensitivity for SD than for ISEE (SD: 91/142, 64.1% vs. ISEE: 28/66, 42.4%, *p* = 0.004). In contrast, the diagnostic sensitivity of intraoperative specimens was significantly higher in ISEE than in SD (SD: 102/142, 71.8% vs. ISEE: 59/66, 89.4%, *p* = 0.007). On the other hand, no difference in the sensitivity of CT-guided biopsy between both groups was observed (SD: 28/50, 56.0% vs. ISEE: 11/20, 55.0%, *p* = 1.0) (Figure 3).

### 3.4. Sensitivity under Ongoing Empiric Antibiotic Therapy

The diagnostic sensitivity of all three procedures combined was significantly higher in SD patients with postoperative TAT than in SD patients with EAT (EAT: 77/89, 86.5% vs. TAT: 53/53, 100%, *p* = 0.004). No such effect was observed in patients with ISEE (EAT: 47/51, 92.2% vs. TAT: 15/15, 100%, *p* = 0.567). Blood cultures, intraoperative specimens and CT-guided biopsies showed no significant difference in SD and ISEE in terms of sensitivity to the timing of antibiotic administration (Table 2). Intraoperative specimen showed the best diagnostic sensitivity in all groups (TAT-SD: 81.1%, TAT-ISEE: 86.7%, EAT-SD: 67.4%, and EAT-ISEE: 90.2%).

### 3.5. Sensitivity of Single Procedure in SD and ISEE Patient Treated with EAT or TAT

The pathogen could always be isolated in the SD- and ISEE-TAT groups, with blood culture, intraoperative specimen, and CT-guided biopsy as single procedure showing no significant difference between SD and ISEE. In contrast, we observed a difference between the SD- and ISEE-EAT groups. The diagnostic sensitivity of blood culture alone was higher in the SD-EAT than in the ISEE-EAT (SD-EAT: 14/89, 18.2% vs. ISEE-EAT: 1/51, 2.1%, *p* = 0.010). The ISEE-EAT group showed better sensitivity than SD-EAT for intraoperative specimen (SD-EAT: 19/89, 24.7% vs. ISEE-EAT: 24/51, 51.1%, *p* = 0.002). CT-guided biopsy revealed no difference between the two groups (SD-EAT: 2/89, 2.6% vs. ISEE-EAT: 0/51, 0.0%, *p* = 0.534) (Table 3).

### 3.6. The First Result of Detected Pathogen in SD and ISEE Treated with EAT or TAT

The first result of pathogen detection from blood culture, intraoperative specimen, and CT-guided biopsy showed no significant differences between SD and ISEE treated with TAT, but pathogens were always detected. The result of the first pathogen isolation in the EAT group was different between ISEE and SD. Blood culture in SD (SD-EAT: 50/89, 64.9% vs. ISEE-EAT: 18/51, 38.3%) and intraoperative specimen in ISEE (SD-EAT: 24/89, 31.2% vs. ISEE-EAT: 28/51, 59.6%, *p* = 0.008) played the most important role. CT-guided biopsy showed no differences between the two groups (SD-EAT: 3/89, 3.9% vs. ISEE-EAT: 1/51, 2.1%) (Table 4).

### 3.7. The role of Each Procedure in Pathogen Detection in Both Entities

To determine which procedure as stand-alone was able to detect the most pathogens, we analyzed pathogen detection for all procedures in both subgroups, considering each procedure separately and in combination with others.

#### 3.7.1. Spondylodiscitis

**Figure 4:**
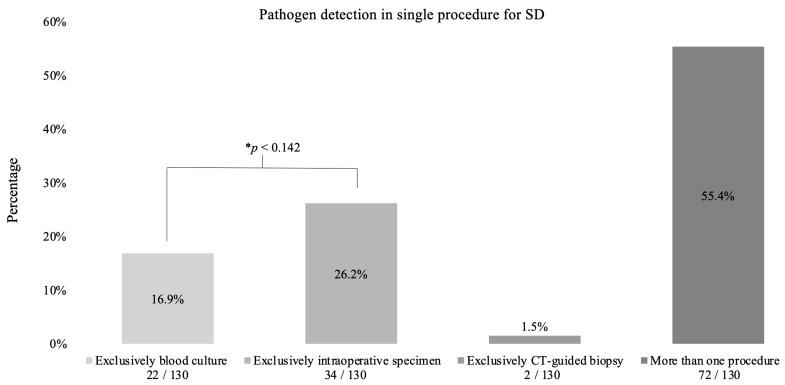
Pathogen detection in spondylodiscitis. This diagram shows in which procedures pathogens were detected in spondylodiscitis patients. There were 142 patients in total. Pathogens were detected in 130 patients. * Binomial test.

A quarter of pathogens were detected exclusively by intraoperative specimens (34/130, 26.2%), while 16.9% (22/130) were identified solely by blood cultures (*p* < 0.142). Only 1.5% (2/130) were detected by CT-guided biopsy. Over half of the pathogens (72/130, 55.4%) were found in more than one procedure (Figure 4).

#### 3.7.2. Isolated Spinal Epidural Empyema

**Figure 5:**
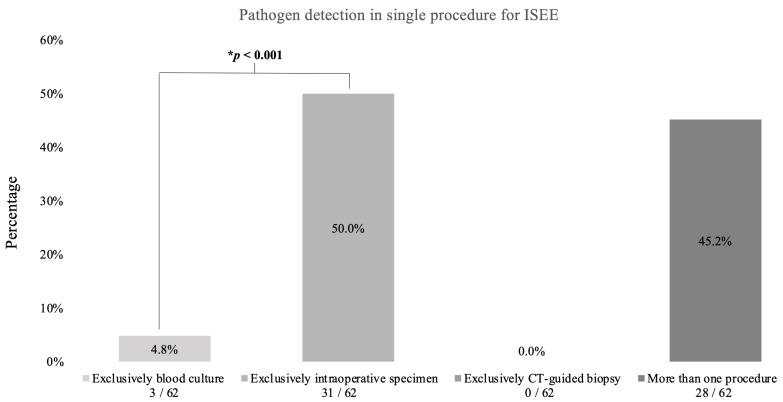
Pathogen detection in isolated spinal epidural empyema. This figure demonstrates the procedures used to detect the pathogens in epidural empyema patients. In total, there were 66 patients. Pathogens were detected in 62 patients. * Binomial test.

Half of the pathogens (31/62, 50%) were exclusively detected by intraoperative specimens, while only 4.5% (3/62) were detected by blood cultures (*p* < 0.001) and none were detected by CT-guided biopsy. Less than half of the pathogens were detected in more than one procedure (28/62, 45.2%) (Figure 5).

### 3.8. The First Result of Antibiogram and Resistogram from All Procedures

The time required to detect pathogens in blood cultures from blood and for intraoperative specimens from pus, tissue, or bone is different. In clinical practice, the results of all procedures (CT-guided biopsy, intraoperative specimen, and blood culture) are not available at the same time to the physician performing the procedure, however, the first result is the most important and provides the antibiogram and resistogram for initiating and switching antibiotic therapy. In this context, the comparison between intraoperative specimen and blood culture is relevant, since both procedures were performed on the same day in our center. There is a predicted delay in CT-guided biopsy, which was usually performed one or two days after surgery.

#### 3.8.1. Spondylodiscitis

**Figure 6:**
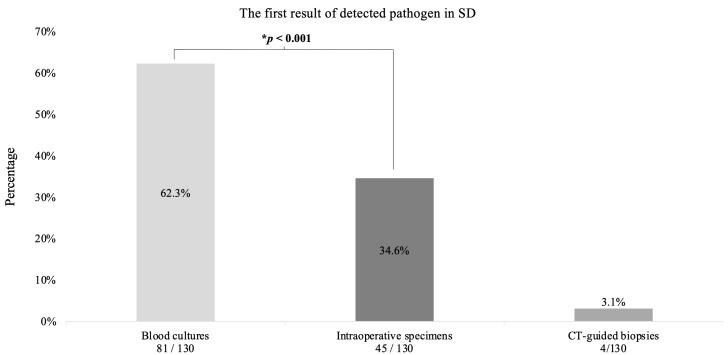
The first result of detected pathogen in spondylodiscitis. This diagram shows the first result of detected pathogen in Spondylodiszitis (SD) patients. * Binomial test.

The first results were obtained in 62.3% (n = 81) from blood cultures, in 34.6% from intraoperative specimens (n = 45) and in 3.1% (n= 4) from CT-guided biopsies (*p* < 0.001) (Figure 6).

#### 3.8.2. Isolated Spinal Epidural Empyema

**Figure 7:**
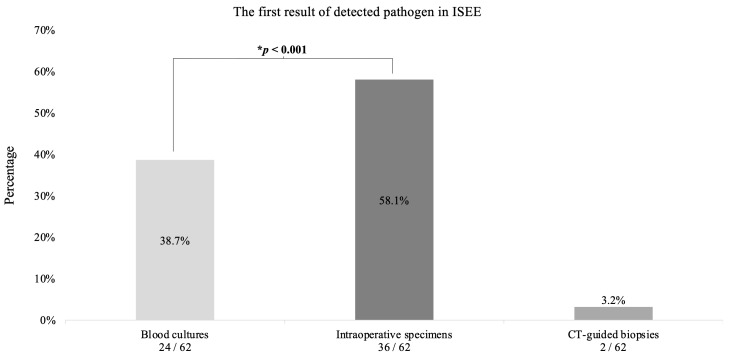
The first result of detected pathogen in isolated spinal epidural empyema. This diagram reveals the first pathogen detection in isolated spinal epidural empyema (ISEE) patients. * Binomial test.

The first results (36 cases) were obtained from intraoperative specimens in 58.1%, from blood cultures in 38.7% (n = 24) and from CT-guided biopsies in 3.2% (n = 2) (*p* < 0.001) (Figure 7).

## 4. Discussion

The main finding of our retrospective observational study is that intraoperative specimens demonstrate the highest diagnostic sensitivity for ISEE, whereas blood cultures show superior sensitivity in detecting pathogens in patients with SD. Interestingly, ongoing antibiotic treatment only affected the sensitivity of these tests in SD patients, with no significant effect on diagnostic yield in ISEE.

Consistent with previous studies, male patients were diagnosed with SD and ISEE twice as often as females in our cohort [5,16]. Gram-positive pathogens were identified in 86.6% of pyogenic infections, which is in line with the literature data ranging from 74 to 82% [21,22,23]. Our antibiotic management was based on the IDSA guidelines [18]. The mortality rate in our cohort was 6%, which is comparable to previous studies [16]. Moreover, our pathogen detection rate of 92.3% using all three diagnostic procedures combined is in accordance with the results of previous observational studies that detected pathogens in 67% and 100% of cases [4,16,24,25]. Authors have reported divergent diagnostic sensitivity of blood culture, image-guided biopsy, and intraoperative specimen for SD and ISEE (Table 5) [4,12,16,17,25,26,27,28,29,30,31,32,33,34,35,36].

The diagnostic sensitivity of blood cultures in SD and ISEE patients varies widely from 30% to 78% according to previously published reports [12,16,17,24,25,26,27,28,30,31,32,33,34,35,36,37,38,39,40]. However, a systematic review of 14 clinical retrospective studies found that blood cultures routinely obtained in 91% of cases had an average sensitivity of 58%, which is consistent with our results (57.2%) [16]. The diagnostic sensitivity of image-guided biopsy ranges from 44.1% to 82.5% and can be increased by using different techniques such as X-ray or CT for sampling and different sites such as psoas, disc, or vertebral body [12,17,24,25,29,36,37,38,39]. Consistent with this spectrum, we were able to detect a pathogen with CT-guided biopsies from psoas abscess in 55.7% of cases.

Intraoperative specimens yielded the best results in the literature ranging from 59.6% to 87.5% [12,17,24,25,26,29,31,34,37,38]. Similarly, using this procedure, we were able to detect a pathogen in 77.9% of patients.

Data on the diagnostic sensitivity of all procedures in SD and ISEE are limited in the literature. The diagnostic sensitivity of blood cultures and intraoperative specimens differed between SD and ISEE. Blood culture sensitivity was higher in SD, whereas intraoperative specimen sensitivity was higher in ISEE. This supports the assumption that SD is mainly a hematogenous dissemination, whereas ISEE is a local encapsulated mass with pus or inflammatory tissue, which can ideally be reached by intraoperative sampling.

Previous studies have shown that blood cultures can detect pathogens in 57.2% of cases, with even higher rates up to 70% in antibiotic-naive patients [4,12,16]. In our study, the sensitivity of all three methods was significantly lower in SD patients under ongoing EAT than in SD patients without antibiotics, whereas the sensitivity in ISEE showed no significant difference in relation to antibiotic therapy.

The diagnostic sensitivity of the stand-alone procedure showed no differences between SD and ISEE when the patient was treated with TAT, whereas the EAT group differed in terms of blood culture as the most sensitive stand-alone procedure in SD and intraoperative specimen in ISEE. Our study also showed the same results in terms of the first result of pathogen detection in SD and ISEE patients treated with TAT or EAT.

In our cohort, pathogen detection in ISEE was higher in intraoperative specimens exclusively compared with blood cultures (50% versus 5%), demonstrating the importance of surgical sampling in ISEE. This was not significant in SD. The first decisive result of pathogen detection in SD patients was achieved in 62.3% of cases by blood culture and in 58.1% of cases of ISEE by the intraoperative specimen in our study. Therefore, blood cultures in SD and intraoperative samples in ISEE seem to be of the highest importance to allow the administration of targeted antibiotics.

CT-guided biopsy was performed only in the psoas abscess but not in the disc compartment in SD or in the epidural empyema in ISEE, where infectious processes usually occur, which may have influenced our results. The results of a CT-guided biopsy for pathogen detection varies and depends on the examination technique, the examiner, patient collective, and the specimen being collected. Numerous other factors, including laboratory parameters (C-reactive protein > 50 mg/L), CT features (nonsclerotic endplate erosions), and magnetic resonance criteria (paravertebral/epidural abscess formation), appeared to be associated with positive pathogen detection [41].

For specific anatomic structures, such as the psoas, CT-guided biopsy with drainage is essential because of the percutaneous minimally invasive technique and success rate in targeting, as well as the low complication rate. In addition, CT-guided biopsy is an essential procedure in patients without instability or neurologic deficits due to the avoidance of surgery with potential complications despite the anesthetic and healing disruption risks. The above demonstrates how important CT-guided biopsy is for the treatment of SD and ISEE. The poorly presented results of CT-guided biopsy in this study compared with blood culture and intraoperative specimen may be influenced by the selective surgical patient, timing of the procedure, and anatomic structure of the collection (psoas abscess only). Nevertheless, this study demonstrated favorable diagnostic sensitivity for CT-guided biopsy compared with the existing literature, which suggested a successful pathogen detection rate ranging from 28.1% to 57.1% [42,43], and provided a novel comparison of all three procedures with limitations.

### Limitations and Strengths of This Study

This study has inherent limitation due to its retrospective nature. However, our findings provide valuable insights into the diagnostic sensitivity of blood cultures, intraoperative specimens, and CT-guided biopsies and serve a basis for prospective research. The monocentric design of our study limits the generalizability of our observations. Nevertheless, our cohort of SD and ISEE patients underwent comprehensive phenotyping, including, detailed demographic, clinical, radiological, laboratory and microbiological assessments, which enhances the internal validity of our data. To confirm our findings and assess their external validity, well-designed multicentric studies are needed, preferably in a prospective interventional setting [44].

## 5. Conclusions

To achieve the best diagnostic sensitivity for causative pathogens, all procedures are essential, particularly intraoperative specimens and blood cultures. Intraoperative specimens yielded the highest single result, followed by blood cultures. However, blood cultures remain crucial due to their non-invasive nature. Blood cultures are crucial for identifying pathogens in SD, while intraoperative specimens are essential for isolating pathogens in ISEE.

Blood cultures play the most important role in pathogen identification in SD, while intraoperative specimens are leading in the description of pathogens in ISEE. Without surgical intervention, nearly half of the pathogens in ISEE patients cannot be detected. This study reveals that the combined diagnostic procedures have lower sensitivity in SD patients with ongoing EAT, but not in ISEE patients.

## Figures and Tables

**Table 1:** Baseline characteristics.

Baseline Characteristics	N = 208	Percentage
Male	136	65.4%
Female	72	34.6%
Age	68 [23–90] y *	-
Spondylodiscitis	142	68.3%
Isolated spinal epidural empyema	66	31.7%
Surgery	208	100%
Blood cultures	208	100%
Psoas abscess	128	61.5%
CT-guided biopsy of Psoas	70/128	54.7%
Known causative pathogens	192	92.3%
Unknown causative pathogens	16	7.7%
Pyogenic spinal infection	187/192	97.4%
Non-pyogenic spinal infection	5/192	2.6%
Gram-positive pathogens	162/187	86.6%
Gram-negative pathogens	25/187	13.4%
Empiric antibiotic therapy	140	67.3%
Targeted antibiotic therapy	68	32.7%
Duration of intravenous antibiotics	4 [3–6] w *	-
Duration of antibiotics	8 [6–12] w *	-
Death	12	5.8%

CT: computer tomography, *: median [interquartile range].

**Table 2:** Diagnostic sensitivity under ongoing empiric antibiotic therapy.

Infection Subgroup	All Three Procedures	Blood Cultures	Intraoperative Specimens	CT-Guided Biopsies
SD with TAT	53/53 (100%)	35/53 (66.0%)	43/53 (81.1%)	11/22 (50%)
SD with ongoing EAT	77/89 (86.5%)	56/89 (62.9%)	60/89 (67.4%)	17/28 (66.7%)
*p*-value *	**0.004**	0.722	0.084	0.568
ISEE with TAT	15/15 (100%)	7/15 (46.7%)	13/15 (86.7%)	4/5 (80%)
ISEE with ongoing EAT	47/51 (92.2%)	21/51 (41.2%)	46/51 (90.2%)	7/15 (46.7%)
*p*-value *	0.567	0.771	0.653	0.319

ISEE: isolated spinal epidural empyema, SD: spondylodiscitis, CT: computer tomography, EAT: empiric antibiotic therapy, TAT: targeted antibiotic therapy, *: Fisher exact test. Bold values are significant results (*p* < 0.05) as indicated in the methods.

**Table 3:** Diagnostic sensitivity of single procedures in SD and ISEE patient treated with EAT or TAT.

Procedure of Pathogen Detection	TAT			EAT		
	SD	ISEE	*p*-Value *	SD	ISEE	*p*-Value *
Exclusively blood culture	8/53 (15.1)	2/15 (13.3)	1.0	14/89 (18.2%)	1/51 (2.1%)	**0.010**
Exclusively intraoperative specimens	15/53 (28.3)	7/15 (46.7%)	0.218	19/89 (24.7%)	24/51 (51.1%)	**0.002**
Exclusively CT-guided Biopsy	0/53 (0.0%)	0/15 (0.0%)	---	2/89 (2.6%)	0/51 (0.0%)	0.534
More than one procedure	30/53 (56.6%)	6/15 (40.0%)	0.380	42/89 (54.5%)	22/51 (46.8%)	0.725
Unknown pathogens	0	0		12	4	

ISEE: isolated spinal epidural empyema, SD: spondylodiscitis, CT: computer tomography, EAT: empiric antibiotic therapy, TAT: targeted antibiotic therapy, *: Fisher exact test. Bold values are significant results (*p* < 0.05) as indicated in the methods.

**Table 4:** The first result of detected pathogen in SD and ISEE treated with EAT or TAT.

Procedure of First Pathogen Detection	TAT			EAT		
	SD	ISEE	*p*-Value *	SD	ISEE	*p*-Value *
Blood culture	31/53 (58.5%)	6/15 (40%)	0.340	50/89 (64.9%)	18/51 (38.3%)	**0.008**
Intraoperative specimens	21/53 (39.6%)	8/1 (53.3%)		24/89 (31.2%)	28/51 (59.6%)	
CT-guided Biopsy	1/53 (1.9%)	1/15 (6.7%)		3/89 (3.9%)	1/51 (2.1%)	
Unknown pathogens	0	0		12	4	

ISEE: isolated spinal epidural empyema, SD: spondylodiscitis, CT: computer tomography, EAT: empiric antibiotic therapy, TAT: targeted antibiotic therapy, *: Fisher exact test. Bold values are significant results (*p* < 0.05) as indicated in the methods.

**Table 5:** References on sensitivity of blood culture, intraoperative specimen, image-guided biopsy.

Author	Blood Culture	Image-Guided Biopsy	Intraoperative Specimen
Vettivel et al. [37]	37 (48.7%)	30/40 (75%) *	
Heuer et al. [38]	145/307 (47%)	213/307 (64%) *	
Widdrington et al. [25]	40/78 (51%)	21/29 (72%)	25/38 (66%)
Hasan et al. [39]	17/40 (42.5%)	33/40 (82.5%)	NR
Stangenberg et al. [34]	97/182 (53.3%)	NR	134/202 (66.3%)
Nolla et al. [12]	46/64 (63.4%)	11/21 (52%)	15/20 (75%)
Colmenero et al. [28]	52/152 (34.2%)	NR	NR
Pigrau et al. [40]	71/91 (78%)	NR	NR
McHenry et al. [17]	156/255 (61.2%)	86/124 (69.4%)	88/113 (77.9%)
Patzakis et al. [33]	13/26 (50%)	NR	NR
Zarrouk et al. [36]	14/29 (48.3%)	11/15 (73.4%)	NR
Carragee et al. [27]	66/111 (59.5%)	NR	NR
Ledermann et al. [30]	25/41 (61%)	NR	NR
Bateman et al. [26]	23/52 (44.2%)	NR	24/32 (75%)
Torda et al. [35]	10/16 (62.5%)	NR	NR
Osenbach et al. [32]	12/40 (30%)	NR	NR
Hadjipavlou et al. [29]	NR	19/26 (73.1%)	25/40 (62.5%)
Nather et al. [31]	5/9 (55.6%)	NR	14/16 (87.5%)
Current study	119/208 (57%)	39/70 (56%)	162/208 (78%)

NR: not reported. *: The authors did not distinguish between image-guided biopsy and intraoperative specimen.

## Data Availability

The original contributions presented in the study are included in the article, further inquiries can be directed to the corresponding author.

## Acknolegdment

We would like to thank Gabriele Schackert and Kay Engellandt for their support.
